# MSFT-YOLO: Improved YOLOv5 Based on Transformer for Detecting Defects of Steel Surface

**DOI:** 10.3390/s22093467

**Published:** 2022-05-02

**Authors:** Zexuan Guo, Chensheng Wang, Guang Yang, Zeyuan Huang, Guo Li

**Affiliations:** 1 School of Modern Post, Beijing University of Posts and Telecommunications, Beijing 100876, China; gzx152@bupt.edu.cn (Z.G.); liguo@bupt.edu.cn (G.L.); 2 School of Artificial Intelligence, Beijing University of Posts and Telecommunications, Beijing 100876, China; yang@bupt.edu.cn; 3 Teaching Affairs Office, Beijing University of Posts and Telecommunications, Beijing 100876, China; huangzeyuan@bupt.edu.cn

**Keywords:** steel surface, detected defects, MSFT-YOLO, YOLOv5, TRANS

## Abstract

With the development of artificial intelligence technology and the popularity of intelligent production projects, intelligent inspection systems have gradually become a hot topic in the industrial field. As a fundamental problem in the field of computer vision, how to achieve object detection in the industry while taking into account the accuracy and real-time detection is an important challenge in the development of intelligent detection systems. The detection of defects on steel surfaces is an important application of object detection in the industry. Correct and fast detection of surface defects can greatly improve productivity and product quality. To this end, this paper introduces the MSFT-YOLO model, which is improved based on the one-stage detector. The MSFT-YOLO model is proposed for the industrial scenario in which the image background interference is great, the defect category is easily confused, the defect scale changes a great deal, and the detection results of small defects are poor. By adding the TRANS module, which is designed based on Transformer, to the backbone and detection headers, the features can be combined with global information. The fusion of features at different scales by combining multi-scale feature fusion structures enhances the dynamic adjustment of the detector to objects at different scales. To further improve the performance of MSFT-YOLO, we also introduce plenty of effective strategies, such as data augmentation and multi-step training methods. The test results on the NEU-DET dataset show that MSPF-YOLO can achieve real-time detection, and the average detection accuracy of MSFT-YOLO is 75.2, improving about 7% compared to the baseline model (YOLOv5) and 18% compared to Faster R-CNN, which is advantageous and inspiring.

## 1. Introduction

With the increasing development of artificial intelligence technology, there are more applications in the industry that incorporate it. Computer vision methods, such as object detection, are now widely being used in the task of detecting material surface defects [1]. In the process of workpiece manufacturing, surface defects in the material will reduce the strength of the material, thus shortening the service life of the workpiece and affecting the quality. However, these problems can be avoided if the material is inspected for defects before processing. Therefore, automated and accurate object detection algorithms play a very important role in the scenario of workpiece manufacturing.

**Figure 1:**
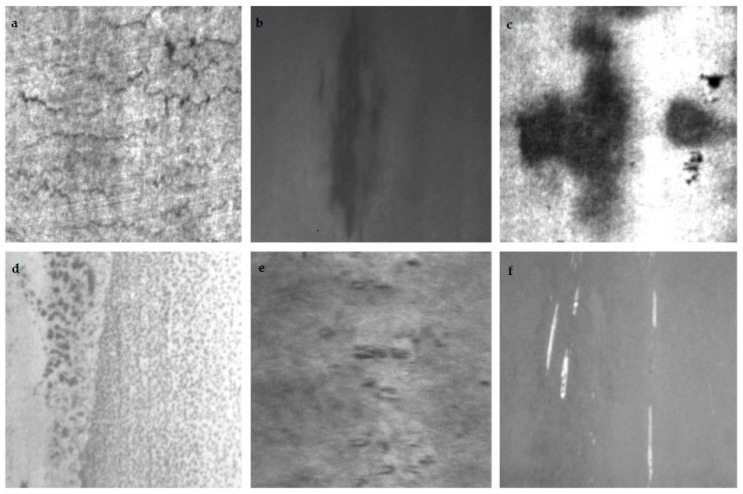
Different types of steel surface defects. (**a**) Crazing, (**b**) inclusion, (**c**) patches, (**d**) pitted surface, (**e**) rolled-in scale, (**f**) scratches.

In the field of computer vision, CNNs (convolutional neural networks) have become the dominant model for vision tasks since 2012 [2]. As a hot topic in computer vision, object detection algorithms can be divided into candidate region-based target detectors (two-stage) and single-target detectors (one-stage). The representative algorithms of the one-stage detector are the YOLO (you only look once) series [3,4,5,6], and the representative algorithms of the two-stage detector are the R-CNN (regions with CNN features) series [7,8,9]. In general, modern detectors use pure convolution network to extract features. Classical image classification networks, such as VGG [10] and ResNet [11], are used as the backbone of the state-of-the-art detectors Faster R-CNN and RetinaNet [12]. The YOLO series, as a representative of the one-stage detector, adopts a newfangled residual network called the Darknet, which allows better feature extraction. However, as shown in Figure 1, the images for defect detection have obvious features, such as large-scale variation in images, more background interference, difficulty to distinguish defects, etc. It is very difficult to solve such problems with the common convolutional-network-based object detection methods, such as the YOLO series.

As increasingly efficient structures emerge and computer vision and natural language processing increasingly converge, the use of Transformer [13] for vision tasks has become a new research direction to reduce the complexity of structures and explore scalability and training efficiency. Transformer models have obtained relatively ideal results in tasks such as object detection and image classification. For steel surface images, to improve semantic discrimination and alleviate category confusion, collecting information from neighboring regions and associating the collections could be beneficial to learn the relationship between objects. In contrast, convolution operation has limitations because a convolution kernel usually only models and computes the relationship between neighboring pixels. In this respect, Transformer models can make up for this flaw of convolution very well.

Actually, the harsh production environment often leads to workpieces with complex backgrounds, such as castings. In order to solve the problems of cluttered defect locations and large-scale variations in defect sizes in the defect images, the object detector should have a strong autonomous adaptation and dynamic adjustment capability. The study in the literature [14] shows that the structure of Transformer is more efficient and flexible. Compared to CNNs, Vision Transformer (ViT) models are much more robust to object interference and region size variation in images. Therefore, our work enhances the performance of the detector by adding the Transformer layer to the backbone and neck to obtain more layers of feature and contextual information in the process of feature extraction and fusion.

On the other hand, the sizes of defects in steel surface images vary greatly, and it is difficult for convolutional neural networks to represent the features of some small objects properly, limited by the feature mapping of a single layer. Therefore, it is essential to seek a more effective way to represent these features. The feature pyramid structure is a common approach. In the convolution process, large objects have more pixel points, while small objects have fewer pixel points. As the convolution layer goes deeper, the features of large objects are easily retained when the features of small objects are easily ignored, so it is difficult to detect small objects in object detection. The feature pyramid could have a variety of feature layers with high semantic content by continuously downsampling the feature points and, afterward, resampling these feature layers, making the width and height of the feature layer large again, and using a large-size feature map to detect small objects. In this aspect, learnable weights is introduced to the path-aggregation neck in order to understand the importance of different input features while applying top-down and bottom-up multi-scale feature fusion repeatedly.

Based on the analysis above, this paper proposes a hybrid detector MSFT-YOLO for the steel surface defect detection based on the idea of the one-stage detector. The model integrates the CSPDarknet [6] and Transformer modules and enlarges the receptive field to predict the multiple scale defects by extending the receptive field of convolution, taking into account the local information of the object while also incorporating the global information. BiFPN (bi-directional feature pyramid network) was also deployed to fuse features at different scales and combine features at different scales to detect the small objects that are difficult to detect. Not only that: we also implement an effective strategy called the multi-step training method to collect defect-free samples from the detection process as a new class of samples and put them into the training of the model so that the multi-step training method reduces the probability of false detection and enriches the training set at the same time. Our experiments show that the improved MSFT-YOLO network structure can reach 75.7 mAP on the NEU-DET dataset, which is able to maintain the detection accuracy while maintaining the detection speed.

In summary, the main contributions of this paper are:

1. The TRANS module is proposed and introduced in the original backbone and neck of YOLOv5, which extends the reception domain of convolution layers and provides more levels of features for detection by combining global information;
2. A weighted bi-directional feature pyramid network is combined in the original model for fusing information at different scales;
3. An effective strategy, including dataset expansion and multi-step training schemes, is used to perform defect detection at high speed with high accuracy on the NEU-DET dataset, demonstrating that the model is superior to other defect detection methods on this mission.

In this paper, we propose a steel surface defect detector called MSFT-YOLO based on YOLOv5. MSFT-YOLO uses a combination of some current techniques in computer vision, including Transformer encoder block, multi-level feature fusion, dataset expansion, and some training techniques. Comparison experiments were designed to verify the effectiveness of the algorithm. The experimental results show that the model can achieve high detection accuracy while also having the ability to detect in real time. The test results on the NEU-DET dataset show that the mAP of MSFT-YOLO is 75.2, while the FPS (frames per second) is 30.6, improving about 7% compared to the baseline model (YOLOv5), which can solve the problem of poor detection of steel surface defects in industrial scenes for images with strong background interference, large changes in defect scale, a large number of small defects, and easily confused defect targets.

## 2. Related Work

### 2.1. General Object Detection

Object detection algorithms have undergone a rapid development phase. As the performance of hand-designed features in traditional object detection is saturated, object detection reaches a plateau. In 2012, Krizhevsky proposed AlexNet to achieve excellent results in image classification, and researchers started to focus on the topic of deep neural networks and apply them to object detection [15].

Deep learning techniques enable object-specific features to be learned directly from data, and this disruptive approach has gained a great deal of attention since 2012 as soon as it was proposed. With the development of deep learning, object-detection-related algorithms are increasingly proposed. Detectors for object detection tasks can be divided into two main categories, two-stage, and one-stage detectors. The two-stage detection framework, which includes a preprocessing step for the region proposal, makes the overall process a two-stage one. Common two-stage detectors include Faster R-CNN, VFNet [16], CenterNet2 [17], and so on. Represented by Faster R-CNN, its network structure contains a feature extractor, classifier, and bounding box regressor, which can be trained end-to-end by a multi-task loss function. Faster R-CNN extracts features from the images by convolutional layer, and the generated feature map is input to both the region proposals network and ROI pooling layer. By combining the classification loss and localization loss in the multi-task loss, Faster R-CNN greatly improves the accuracy in detection. RPN (region proposal network) is an important improvement of Faster R-CNN. In traditional detection, region proposals are usually extracted using sliding window and selective search, which is very time-consuming. However, the input of the RPN network includes images of arbitrary size, and the output is directly available as a series of region proposals [18]. The pixel points are first configured with many different anchor boxes, and the Softmax function is used to classify them and determine whether there is a target in the current anchor box. After that, the anchor box position is corrected by the border regression function, and the exact coordinates of the region proposals are finally obtained. A one-stage detection framework, i.e., a framework without region proposal, does not separate the detection and proposal processes, making the whole process one stage. The common one-stage detectors include YOLOX [19], FCOS [20], DETR [21], Scaled-YOLOv4 [22], etc.

### 2.2. Vision Transformer

Transformer is a deep neural network primarily based on a self-attentive mechanism, which was originally applied in the field of natural language processing. Inspired by the powerful representation capabilities of Transformer, researchers propose to extend Transformer to computer vision tasks. Compared to other networks, such as convolutional and recurrent networks, Transformer-based models show competitive or even better performance. Vision Transformer applies Transformer architecture directly to a series of image blocks for classification tasks, demonstrating that Transformer structures applied to images can also achieve excellent results. Vision Transformer combines knowledge from CV (computer vision) and NLP (natural language processing). Firstly, ViT splits the original images into patches, then flattens them into sequences, which are fed into the encoder part of the original Transformer model and finally into a fully connected layer to classify the images. Suppose the original input image size is *H* × *W* × *C* and the length and width of each image block is *P*. Then, the number of patches *N* is $HW/P^{2}$. Each image patch is expanded into a one-dimensional vector, and the size of each vector is *P* × *P* × *C*, so the final input is $N\times \left(P^{2}\cdot C\right)$. Then, a randomly initialized position encoding is introduced so that the features used for classification are obtained.

DETR (Detection Transformer) is a very successful method of applying Transformer in the field of object detection. The overall structure of DETR is similar to Transformer; firstly, the features acquired by backbone are tiled and then sent to encoder after adding position information to obtain the candidate features and finally decoded by decoder in parallel to obtain the final detection result.

### 2.3. Multi-Scale Feature Fusion

Feature fusion is an important problem in the object detection task. Because the size of different objects varies during the convolution calculation, the scales of features extracted in the backbone are different; using such features for prediction leads to problems, such as difficult detection of small objects and missing feature information. Making full use of features of different scales in the prediction can greatly improve the efficiency of detection. The feature pyramid network (FPN) [23] method is proposed to alleviate the above problem by balancing the features of different-size objects through the operation of down-sampling and up-sampling of feature map. In Faster R-CNN, FPN downsamples the features four times. Although the deep-level features abstract the images well and enhance the classification performance of the network, for some small targets with few pixel points, the features are severely lost after down-sampling, so the problem of poor detection of small objects occurs in the detection. Continuing the idea of FPN, a more efficient multi-scale fusion approach is proposed in EfficientDet [24], which better balances the feature information at different scales by introducing the concept of weights. Currently, common path aggregation blocks include FPN, PANet [25], NAS-FPN [26], BiFPN, ASFF [27], etc. This weighted bidirectional feature pyramid network achieves simple and fast multi-scale feature fusion.

### 2.4. YOLO

The one-stage detector usually contains three main components, namely the backbone, neck, and head, where backbone is a convolutional neural network that can receive images of different sizes and form the overall features of the image, neck represents a series of network layers that can fuse the image features extracted by backbone according to certain laws to make the feature semantic information richer and output the processed features as the input of the prediction layer, and, finally, head predicts the input features, and the classifier obtains the class of objects and generates the final coordinates of the bounding box.

The YOLO series is a typical representative of one-stage detectors. YOLO uses an end-to-end neural network on a whole image, where the network divides the image into grid regions while predicting the rectangular boxes in each region of the grid, treating the object detection problem as a regression problem where the model only needs to perform one operation on the input. Each grid is responsible only for the targets whose centroids are within the grid, and each grid predicts the coordinates of several rectangular boxes as well as their scores. Each rectangular box obtained in the end corresponds to a five-dimensional output, i.e., coordinates and confidence level. Such an approach is fast in inference but less accurate.

YOLOv5 currently has significant advantages in speed and accuracy, with important modules including Focus, CSPbottleneck, SPP, and PANet. The backbone is selected as CSPDarknet53, the structure of CSPDarknet53 consists of several residual networks stacked together, and there are a series of Resblock-body modules in the residual network; each Resblock-body undergoes one down-sampling and multiple residual networks, where the activation function of the convolution layer is the Mish. YOLOv5 uses PANet as the neck of the model, and the input is the feature map output from the backbone, which is feature-fused to obtain features with richer semantic information to be sent to the head for detection. The PANet structure is improved based on FPN in terms of feature extraction, which not only conveys semantic information but also locational information. A bottom-up pyramid is added to the FPN structure to pass the strong localization features from the lower layer to the upper layer, which is achieved to complement the FPN feature fusion.

## 3. Proposed Method

In this section, we provide a general discussion of the model architecture of MSFT-YOLO. Our proposed network, MSFT-YOLO, is based on CNNs and Transformer, and the overall network structure is built based on YOLOv5l. As a representative method of one-stage detectors, the YOLO series has always maintained the characteristics of accuracy and efficiency. YOLOv5, as the latest solution of the YOLO series, is used in many industrial scenarios. YOLOv5 has four different models, including YOLOv5s, YOLOv5m, YOLOv5l, and YOLOv5x. Typically, YOLOv5 uses the architecture of CSPDarknet53, with SPP layer as the backbone, PANet as neck, and YOLO detection head. In our experiments, the mAP of YOLOv5l is ahead of YOLOv5s and YOLOv5m 2.5% and 1.8%, and the FPS reaches 52.5, which has the potential of real-time detection and is easy to deploy in real applications, so YOLOv5l is finally chosen as the baseline for further improvements.

### 3.1. MSFT-YOLO

For steel surface images with large scale variance and complex scenes, to improve the semantic discriminability and alleviate global category confusion, it is necessary to collect and associate scene information from large neighborhoods to learn the relationship between objects. However, for convolutional networks, the locality of convolution operations limits their ability to obtain global contextual information. In comparison, Transformer models are capable of a genuinely global focus on the dependencies between image feature blocks and retain sufficient spatial information for object detection. On the other hand, in the process of industrial inspection, both accuracy and speed of detection are important. BiFPN achieves effective bi-directional cross-scale connectivity and weighted feature fusion by optimizing the multi-scale feature fusion method, which balances the efficiency and accuracy of the model. To improve the scalability of the model and the ability to capture contextual information, we propose the MSFT-YOLO model.

**Figure 2:**
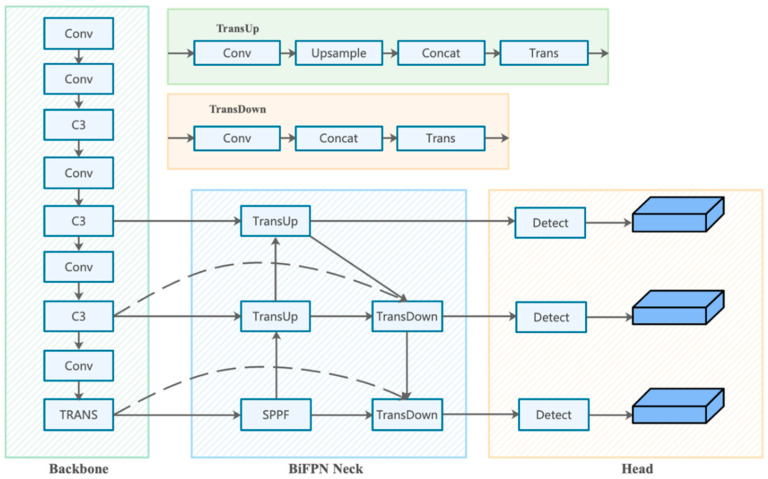
The whole network structure. (1) CSPDarknet53 backbone with a TRANS module at the end. (2) Neck using the structure of the BiFPN. (3) Feature map of three detection heads using the TRANS module in neck.

The overall schematic of MSFT-YOLO is shown in Figure 2, which mainly contains three parts: the backbone network part, the feature enhancement part, and the prediction part. In the first part, backbone, instead of using the original convolution layers of YOLOv5,we mainly use the self-developed TRANS structure, which extends the reception domain of convolution by assembling it into CSPDarknet. TRANS provides multi-level features with global information for detection, which also enhances the ability of MSFT-YOLO to recognize the background features on the steel surface. In the neck of the network, we replace PANet with a simple but effective BiFPN structure to weight the combination of multi-level features of backbone, and we explore the prediction potential of self-attention based on YOLOv5 by integrating the TRANS module into the prediction heads instead of the original prediction heads, which can accurately localize objects in high-density scenes and can handle large scale variance of objects. The specific details of TRANS are described in Section 3.2.

By observing the collected steel defect surface images, it can be found that the defect samples are characterized by a large number of defects and a huge difference in the size of different defects due to the industrial production environment. Moreover, the defect backgrounds are often very heterogeneous, but the correlation between different types of defects and the background near the defects is strong. Our proposed network, MSFT-YOLO, developed with YOLOv5l as the baseline, incorporating the TRANS module and BiFPN in the backbone and neck structure, makes the detection process more feature-rich by combining the local and global features of the object to achieve the best detection results.

As shown in Figure 2, in the backbone part of MSFT-YOLO, we only add the TRANS module at the end; this is because, after experiments, when adding TRANS module in other stages of backbone, since the network is still relatively shallow, adding TRANS module will trigger boundary regression, thus losing some contextual information that is helpful to judge. The resolution of the feature map at the end of the backbone is low, which means TRANS can be applied to images with low-resolution features to reduce the storage cost of the model to a certain extent, and the computational cost can be reduced when applying the model, which is more suitable for deployment and usage in industrial scenarios. In the neck part of the model, due to the small size of the dataset images themselves, replacing the original PANet with BiFPN can better combine the high-resolution and low-resolution image feature information, especially for crazing and rolled-in scale, two types of defect images with a large number of small objects, which plays a great role. Moreover, after replacing the convolution module in CSPbottleneck with the TRANS module, the overall computation of the model becomes larger and the features are more complex, but the detection of features with cluttered backgrounds is greatly improved.

### 3.2. TRANS

CNN networks have been a very common scheme in object detection, and they are very effective in extracting the underlying features and visual structures of images. These underlying features obtained by CNN constitute the key points, lines, and some basic image structures in the patch level. Such underlying geometric features of the image can abstract the image features well and often focus on the consistency under transformations, such as translation and rotation. However, after detecting these basic visual elements, the high-level visual semantic information is more concerned with how these fragmented elements form a whole. At present, it seems that Transformer has a better effect in dealing with the relationship of these elements. Combining the features of CNNs and Transformer, TRANS is able to achieve better results by using the advantages of CNNs in extracting the underlying vision features and the strength of Transformer in processing the relationship between visual elements and objects.

Affected by the production environment, the steel surface defect images are often characterized by a cluttered and complex background, and, because the processes that cause the same types of defects have similar effects on the steel surface, the range features near the images of the same types of defects behave similarly, while the range features near the defects behave differently for different types of defects. In the task of steel surface defect detection, combining Transformer in the feature extraction stage allows collecting information related to the defect features in a larger neighborhood, which helps the network to learn the relationship between objects and thus improve the accuracy of detection.

**Figure 3:**
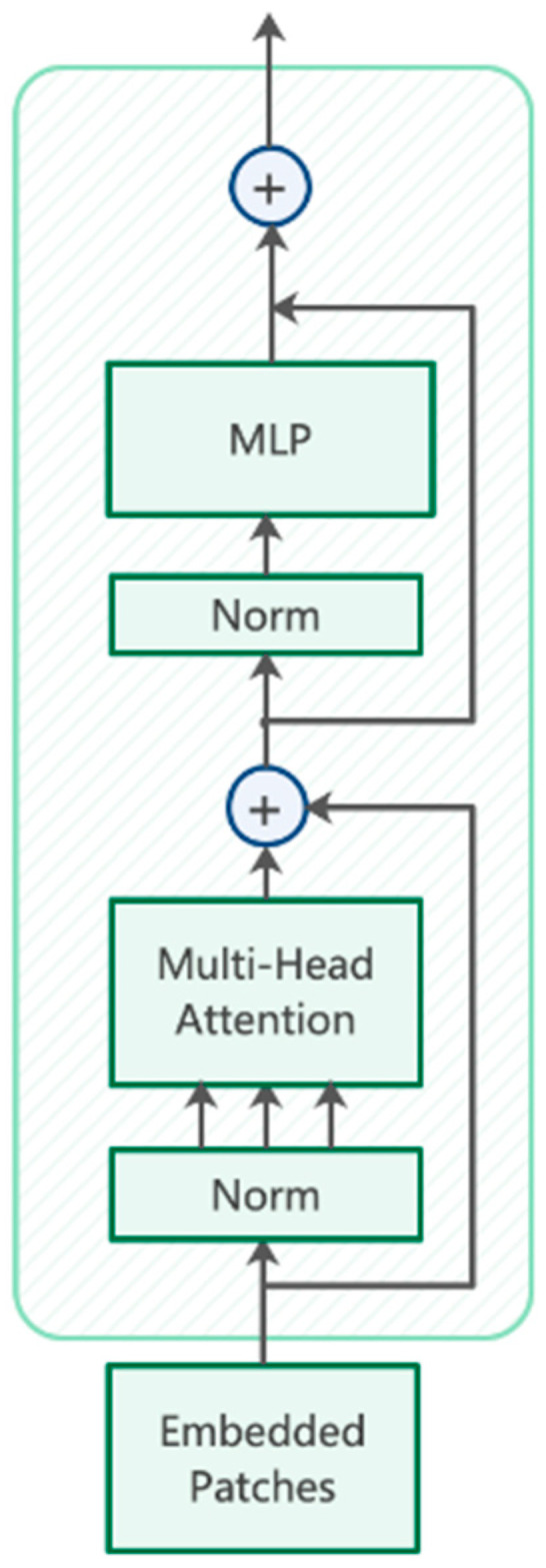
The architecture of TRANS encoder.

In order to make full use of the global information of the defect images, we replaced some convolution blocks and modules of the CSPbottleneck in the original version YOLOv5 with Transformer encoder blocks. The structure of TRANS is shown in Figure 3. Compared with the original structure, we think the TRANS module can cover the global information better and provide richer defect contextual features. Each TRANS module consists of two sub-layers, the first one being a multi-headed attention layer and the second one being a fully connected layer. In general, the TRANS module makes full use of the self-attentive mechanism while exploring the feature representation potential, adding the ability to capture different positional information to the overall model. On the NEU-DET dataset, the TRANS module proved to be able to have better performance.

It is worth noting that we only use the TRANS module at the end of the backbone of MSFT-YOLO, which is the main difference from the backbone of CSPDarknet. This is because the Transformer module triggers boundary regression when the network layers are still relatively shallow, thus losing some feature context information. The feature map at the end of the backbone has low resolution, and applying TRANS to images with low-resolution features can reduce the storage cost of the model and the computational cost when using the model, which is more suitable for deployment and use in industrial scenarios.

### 3.3. BiFPN

**Figure 4:**
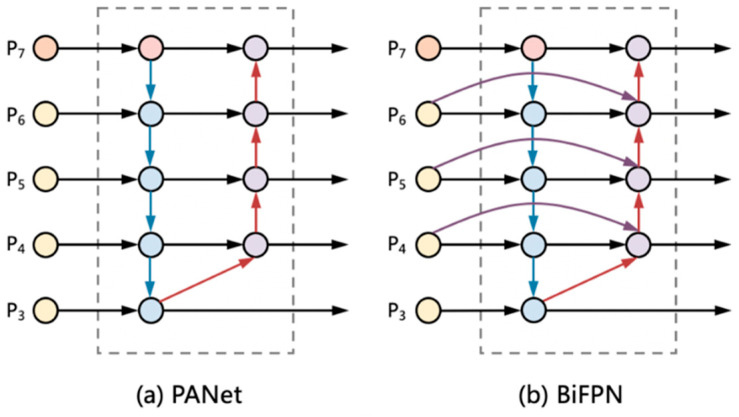
Feature network design (**a**) PANet adds an additional bottom-up pathway on top of FPN. (**b**) BiFPN implements two optimizations for cross-scale connections.

The role of neck part is to make better use of the features extracted by the backbone. By up-sampling, down-sampling, and fusion of the features extracted by backbone at different stages, the model’s ability to detect features at different scales can be significantly improved. As shown in Figure 4a, PANet used in YOLOv5 is an efficient method, and, as the first model proposing a secondary fusion of features from the bottom up, PANet proves the effectiveness of the bidirectional fusion scheme. However, in our experiments, BiFPN, as a complex cross-scale bidirectional fusion method [28,29,30], achieved better results, which works as shown in Figure 4b. Since the individual types of defect images in the dataset used to have fewer pixel points, the shallow features extracted by backbone work better for the recognition of these more obvious defects. When it comes to defects that are difficult to identify, the deeper features are better at abstracting the characteristics of the defects. BiFPN allows the network to understand the importance of each by adding additional weights to the input features of different resolutions, which leads to better integration of different scale features.

When fusing features at different scales, BiFPN does not simply sum or concatenate them but adds weights to the input features of different resolutions. As shown in Figure 4b, on top of PANet, if the original input nodes are at the same level as the output nodes, BiFPN adds extra edges between them to fuse more features without adding too much extra computation. Three weighting strategies are proposed in the paper, unbounded fusion, Softmax-based fusion, and fast normalized fusion; the final strategy used is the third one.

Fast normalized fusion has a very similar learning behavior and accuracy to Softmax-based fusion, which works as shown in Equation (1): *w_i_* is a learnable weight and *w_i_* ≥ 0, which is then passed after Relu to ensure the stability of the value. Eventually, the value of each normalized weight is also between 0 and 1.


(1) $$O=\sum_{i}\frac{w_{i}}{𝜖+\sum_{j}w_{j}}\cdot I_{i}$$

BiFPN integrates bidirectional cross-scale connectivity and fast normalized fusion with learnable weights. As a concrete example, here, we describe the two fused features shown in Figure 4b at layer 6, as shown in the formula below, where ${\vec{P}}^{in}=\left(P_{l_{1}}^{in},P_{l_{2}}^{in},\ldots \right)$, and ${\vec{\;P}}^{in}$ represents the list of input multiscale features and $P_{l_{i}}^{in}$ represents the input features of level $l_{i}$. ${\vec{P}}^{td}=\left(P_{l_{1}}^{td},P_{l_{2}}^{td},\ldots \right)$ represents the list of intermediate features on the path. ${\vec{P}}^{in}$ outputs a new ${\vec{P}}^{out}$ by aggregating a series of different features.

(2) $$P_{6}^{td}=\mathrm{Conv}\left(\frac{w_{1}\cdot P_{6}^{in}+w_{2}\cdot \mathrm{Resize}\left(P_{7}^{in}\right)}{w_{1}+w_{2}+\epsilon }\right)$$

(3) $$P_{6}^{out\;}=\mathrm{Conv}\left(\frac{w_{1}^{\prime }\cdot P_{6}^{in\;}+w_{2}^{\prime }\cdot P_{6}^{td}+w_{3}^{\prime }\cdot \mathrm{Resize}\left(P_{5}^{out\;}\right)}{w_{1}^{\prime }+w_{2}^{\prime }+w_{3}^{\prime }+\epsilon }\right)$$

In the Equation (3), $P_{6}^{td}$ is the intermediate feature of 6 levels in the up-down path, and $P_{6}^{out}$ is the output feature of 6 levels in the bottom-up path. All other features are constructed similarly. It is worth noting that, unlike the original BiFPN proposed in EfficientSet, the SPP add-on module is used in the path aggregation neck to enhance the intermediate features, and the cross-stage partial (CSP) connection is used instead of simple convolution for feature processing.

In the network structure we designed, we explored the role of Vision Transformer for feature fusion in addition to using the BiFPN structure for the neck part. In the two types of defective images of the dataset, crazing, and rolled-in scale, the background is very cluttered. The images have a large number of objects and contain a large number of small objects. As shown in Table 1, after replacing the convolution module in CSPbottleneck with the TRANS module, the overall computation of the model becomes larger and the features are more complex, but the detection of small features with background clutter is greatly improved. Compared with CSPbottleneck, we believe that the TRANS module works better because it can cover more global information and rich contextual information, making full use of the feature representation potential of the self-attentive mechanism.

### 3.4. Data Pre-Processing and Multi-Step Training Method

**Figure 5:**
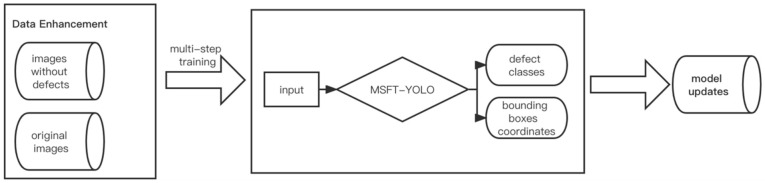
The flow diagram of multi-step training method.

For the mission of steel surface defect detection, we also proposed some useful strategies. During the preprocessing of the dataset, the small size of the dataset with only 1800 images may cause insufficient training and underfitting of the model, so we studied and introduced effective strategies, such as data augmentation and feature enhancement, to help training, including MixUp [31], CutMix [32], and Mosaic. As shown in Figure 5, the model obtained after data pre-processing has false detection for some defect-free samples during the detection process, so we used a multi-step training method to put defect-free samples into the model training as a new class of data, which reduces the probability of false detections and enriches the training set at the same time.

## 4. Experiments

The performance of MSFT-YOLO was evaluated on the NEU-DET dataset, and, after our experiments, the results proved that the design of MSFT-YOLO is reasonable and effective and has the value of practical application in industrial scenarios.

### 4.1. Datasets

**Figure 6:**
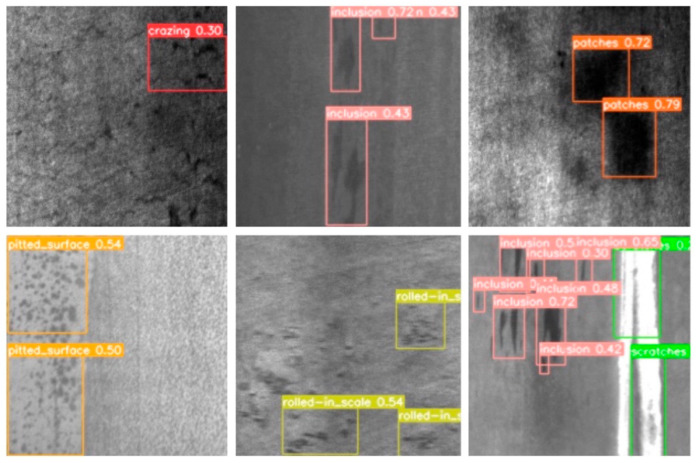
Some visualization results from our MSFT-YOLO on NEU-DET dataset; different categories use bounding boxes with different colors.

The main dataset used in our paper, NEU-DET, is a surface defect database published by Northeastern University that collects six typical surface defects of hot-rolled steel strip, including crazing, inclusion, patches, pitted surface, rolled-in scales, and scratches. The database includes 1800 grayscale images of six different types of typical surface defects, with each type of defect containing 300 samples and each sample image containing multiple defects, with examples of the inspection images shown in Figure 6.

### 4.2. Evaluations Metrics

To validate the performance of MSFT-YOLO, mAP is used as the main measurement metric. The mAP refers to the mean average precision. When evaluating an algorithm, an intersection over union (*IoU*) threshold is set to determine the correct and incorrect detection, and, so long as the *IoU* of the box found by the algorithm is greater than this threshold, it is a valid detection and the result used to calculate mAP is used as the final evaluation index. In particular, mAP is the average of all 10 intersections over the union (*IoU*) thresholds in the range [0.50, 0.95] with a uniform step size 0.05 of all categories, which is used as the primary metric for ranking. The calculation formula is as follows:

(4) $$IoU=\frac{A\cap B}{A\cup B}\;$$

In addition to detection accuracy, another important evaluation criterion for object detection tasks is speed, which plays a crucial role in real-time tasks. The metric that usually judges the speed of object detection is frames per second (FPS), i.e., the number of images that can be processed in each second. It is particularly important to note that the calculation of the FPS metric needs to be completed under the same hardware conditions.

### 4.3. Implementation Details

In the MSFT-YOLO model used for the experiments, as shown in Figure 2, we combined the TRANS structure based on the CSPDarknet as the backbone. For the neck part of the model, we used BiFPN path-aggregation. In the head part of the model, we explored the prediction potential of self-attention based on YOLOv5 by integrating the TRANS module into the prediction heads instead of the original prediction heads. Our model uses SGD as the optimizer, with a weight decay of 0.0005 and momentum of 0.937 as default. For the learning rate update, the warm-up method is used to initialize the learning rate. In the warm-up phase, a one-dimensional linear interpolation is used to update the learning rate for each iteration, and the cosine annealing algorithm is used to update the learning rate after the warm-up phase. In the initial training of the model, we first performed a warm-up training of 3 epochs and the momentum of the optimizer SGD is set to 0.8. After the warm-up training, the initial learning rate is set to 0.02 and the minimum learning rate is 0.01 × 0.1. After that, the model is trained for 200 epochs.

### 4.4. Experimental Results

Table 2 shows the results of our model evaluated on the NEU-DET dataset. In industrial scenarios, not only the accuracy of the object detection task is important but also the detection efficiency is one of the factors to measure whether it can be put into use in industrial scenarios. Only by ensuring both the detect result and the detect speed will the machine make the correct judgment in real time, thus meeting the requirements for use in industrial production. Therefore, in this part of the exposition, both the average mean accuracy (mAP) as a model and the frames per second (FPS) will be used for a comprehensive evaluation of the defect detection model.

**Figure 7:**
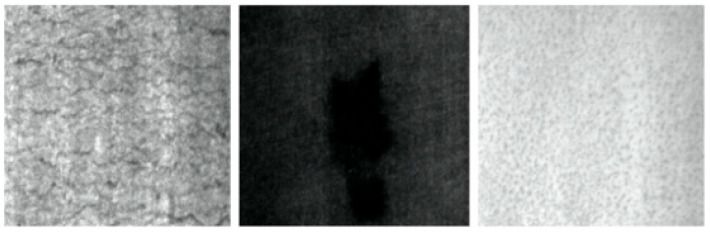
Defect images of crazing, patches, and pitted surface.

As the data in Table 2 show, YOLOv3 has the fastest detection speed but the lowest mAP and the worst capture for small defective objects. Our proposed method based on YOLOv5 has better detection accuracy in all categories than YOLOv5 itself, while the three categories of crazing, patches, and pitted surface, where the object background has the greatest impact on defect detection (as shown in Figure 7), achieve the best results in the two categories of defects with the highest difficulty for human eye recognition, indicating that the design of our scheme works as expected. Although the Faster R-CNN and RetinaNet methods also perform well in some categories of detection, the overall average accuracy is still slightly inferior to our proposed method. The reason for this is that our method better incorporates features at different levels and takes into account the background effects of the detected images. However, for the categories with more obvious defects, the extracted features are more complex, which also allows the background factors to have some negative impact on the features themselves. Combined with the FPS metrics, our method achieves a high detection accuracy along with a high FPS, which has the potential for real-time detection.

### 4.5. Ablation Study

As Table 3 illustrates, it can be observed from the experimental data that, by adding the TRANS module at the backbone, the detection of the two more obvious defects, patches and scratches, is greatly improved, and BiFPN has a greater effect on crazing, patches, pitted surface, rolled-in scale, and scratches, which all have more than 3% bonus. By analyzing the detection results, TRANS enables the model to adapt to a wider range of aspect ratios, solving the problem of samples with uneven distribution of defect aspect ratios. At the same time, since defects often appear in independent and irregularly shaped combinations, the high robustness of TRANS to severe disturbances, perturbations, and area shifts, as well as the ability to integrate high-level visual semantic information, enables the collection of information related to defect features in a larger neighborhood, adding the ability to capture information about different locations to the model.MSFT-YOLO incorporates the TRANS module and BiFPN, which again improves the detection accuracy in two categories, scratches and pitted surface. The BiFPN structure enables the model to adapt to larger variations in defect size, solving the problem of large differences in defect scale distribution. Our method is improved with YOLOv5 as the baseline, and, although the detection speed is reduced by 40%, it still has the potentiality of real-time detection, and the detection accuracy is improved from 0.682 to 0.757, which is a large improvement in detection accuracy. Through the analysis of the detection samples, our inclusion of the TRANS module and the fusion of BiFPN have played a positive role in the model accuracy improvement, and it can be seen that our method has a very obvious effect on the detection of defects in industrial scenes with complex backgrounds and large object scale disparities by combining global features for multi-level feature fusion.

## 5. Conclusions

In this paper, we designed a steel surface defect detector called MSFT-YOLO based on YOLOv5. MSFT-YOLO uses a combination of some current techniques in computer vision, including Transformer encoder block, multi-level feature fusion, dataset expansion, and some training techniques. For the problems of the cluttered background of defect images and easy confusion of defect categories, the TRANS module based on Transformer is proposed to be added to the backbone and detection head. For the problems of large changes in defect scales and poor detection of small defects, the BiFPN structure is proposed, which enhances the adjustment ability of the detector for objects of different scales by fusing features of different scales. By testing on the NEU-DET dataset, MSFT-YOLO achieves 0.752 mAP, a 7.5% improvement over the baseline, while the FPS is 30.6, indicating that the algorithm reaches excellent accuracy and also has the potential for real-time detection, which means it is an object detection algorithm with practical value. In future studies, richer datasets will be introduced to the model to enhance its generalization capability, and the model will be compressed to better adapt to real-time monitoring in industrial scenarios. We have accumulated a great deal of experience in processing steel surface defect datasets and designing detection algorithms during our experiments and hope this article can be useful to more developers and researchers in dealing with steel surface defects.

## Figures and Tables

**Table 1:** The comparison between mAP and model size of different network.

Title 1	Crazing	Rolled-In Scale	mAP	Model Size
BiFPN	0.513	0.521	0.732	85.2 M
BiFPN + TRANS	0.569	0.527	0.743	90.8 M

**Table 2:** The comparison of detecting results on NEU-DET.

Types	YOLOv3	YOLOv5	Faster R-CNN	Retina-Net	OURS
Crazing	0.371	0.513	0.376	0.459	**0.569**
Inclusion	0.580	0.797	0.802	**0.842**	0.808
Patches	0.783	0.905	0.853	0.911	**0.935**
Pitted surface	0.329	0.774	0.815	0.747	**0.821**
Rolled-in scale	0.342	**0.542**	0.540	0.435	0.527
Scratches	0.570	0.563	**0.892**	0.816	0.835
mAP	0.496	0.682	0.713	0.702	**0.752**
FPS	61.2	52.5	24.0	48.2	30.6

In Table 2, the best result of each type of defect is bolded.

**Table 3:** Ablation study on NEU-DET.

Method	Crazing	Inclusion	Patches	Pitted Surface	Rolled-In Scale	Scratches	mAP	FPS
YOLOv5l (baseline)	0.513	0.797	0.905	0.774	0.542	0.563	0.682	52.5
+TRANS (backbone)	0.501	0.816	**0.932**	0.77	0.53	**0.785**	0.722	43.3
+BiFPN	**0.552**	0.791	**0.935**	**0.807**	**0.575**	**0.73**	0.732	41.2
+Multi-step training	**0.561**	0.82	0.933	**0.828**	0.529	**0.838**	0.757	29.1

In Table 3, the data with an improvement of 3% or more compared to the baseline are bolded.

## Data Availability

The data presented in this study are available on request from the corresponding author.
